# CRISPR/Cas9 Promotes Functional Study of Testis Specific X-Linked Gene *In Vivo*

**DOI:** 10.1371/journal.pone.0143148

**Published:** 2015-11-24

**Authors:** Minyan Li, Rui Huang, Xue Jiang, Yuxi Chen, Zhen Zhang, Xiya Zhang, Puping Liang, Shaoquan Zhan, Shanbo Cao, Zhou Songyang, Junjiu Huang

**Affiliations:** 1 Guangdong Province Key Laboratory of Reproductive Medicine, School of Life Sciences and the First Affiliated Hospital, Sun Yat-sen University, Guangzhou, China; 2 State Key Laboratory of Biocontrol, Institute of Healthy Aging Research and SYSU-BCM Joint Research Center, School of Life Sciences, Sun Yat-sen University, Guangzhou, China; 3 Key Laboratory of Reproductive Medicine of Guangdong Province, the Third Affiliated Hospital of Guangzhou Medical University, Guangzhou, China; University of Nevada School of Medicine, UNITED STATES

## Abstract

Mammalian spermatogenesis is a highly regulated multistage process of sperm generation. It is hard to uncover the real function of a testis specific gene *in vitro* since the *in vitro* model is not yet mature. With the development of the CRISPR/Cas9 (Clustered Regularly Interspaced Short Palindromic Repeats/CRISPR-associated 9) system, we can now rapidly generate knockout mouse models of testis specific genes to study the process of spermatogenesis *in vivo*. SYCP3-like X-linked 2 (SLX2) is a germ cell specific component, which contains a Cor1 domain and belongs to the XLR (X-linked, lymphocyte regulated) family. Previous studies suggested that SLX2 might play an important role in mouse spermatogenesis based on its subcellular localization and interacting proteins. However, the function of SLX2 *in vivo* is still elusive. Here, to investigate the functions of SLX2 in spermatogenesis, we disrupted the *Slx2* gene by using the CRISPR/Cas9 system. Since *Slx2* is a testis specific X-linked gene, we obtained knockout male mice in the first generation and accelerated the study process. Compared with wild-type mice, *Slx2* knockout mice have normal testis and epididymis. Histological observation of testes sections showed that *Slx2* knockout affected none of the three main stages of spermatogenesis: mitosis, meiosis and spermiogenesis. In addition, we further confirmed that disruption of *Slx2* did not affect the number of spermatogonial stem cells, meiosis progression or XY body formation by immunofluorescence analysis. As spermatogenesis was normal in *Slx2* knockout mice, these mice were fertile. Taken together, we showed that *Slx2* itself is not an essential gene for mouse spermatogenesis and CRISPR/Cas9 technique could speed up the functional study of testis specific X-linked gene *in vivo*.

## Introduction

Spermatogenesis is a physiological process of spermatozoa production. It is a complex and tightly regulated process which can be divided into three stages: mitosis, meiosis and spermiogenesis [1]. During mammalian meiosis prophase I, X and Y chromosomes are inactivated and compartmentalized into a peripheral nuclear subdomain called the XY body. Many DNA damage response proteins, such as 53BP1, locate at the XY body during meiosis [2]. The components of the XY body are thought to contribute to meiotic sex chromosome inactivation (MSCI). If there is any defect in MSCI, meiotic sterility or aneuploidy might occur [3–5].

Previous study suggested that SYCP3-like X-linked 2 (SLX2, also known as 1700013H16Rik or XLR6) might be involved in MSCI [6]. SLX2 is a member of the XLR (X-linked, lymphocyte regulated) family. The XLR protein family is a group of proteins containing the Cor1 domain, and their coding genes are located on the X chromosome. The XLR family includes more than 40 members [7–10], some of which are involved in spermatogenesis [11–18]. SLX2 was suspected to play an important role in this process since it is expressed in testis during the meiotic prophase I and mainly localized to the XY body [6, 7]. Nevertheless, there is no good model to investigate its real function in spermatogenesis so far.

Genome editing tools enabled site-specific gene editing by inducing double stranded DNA breaks (DSBs). DSBs will be repaired through either error-prone non-homologous end joining (NHEJ) or high-fidelity homologous directed repair (HDR), which will result in indels or precise gene replacement respectively [19]. Indels in a target gene usually lead to frame-shift mutation, which disrupts the target gene expression. Commonly used genome editing tools include meganuclease [20], zinc finger nuclease (ZFN) [21, 22], transcription activator-like effector nuclease (TALEN) [23–26] and the CRISPR/Cas9 system [27, 28]. Among these genome-editing tools, CRISPR/Cas9 system is the most rapidly developed one. CRISPR/Cas9 system, composed of gRNA (guide RNA) and Cas9 endonuclease, can be easily targeted to virtually any gene by designing a new gRNA [29]. The gRNA-Cas9 complex will bind to the target site through RNA-DNA hybridization, leading to activation of Cas9 endonuclease. Then the activated Cas9 endonuclease will cleave the target sites [30, 31]. To target a new gene, we only need to change the 5’ end 20 nt sequence of the gRNA. The ease and high efficiency of CRISPR/Cas9 system make it widely applied in many species [32]. Thanks to the development of CRISPR/Cas9 system, it is a routine method to generate gene-modified founder mouse within 1 month.

Here, we generated *Slx2* knockout mouse model by using CRISPR/Cas9 system to study functions of *Slx2 in vivo*. Two gRNAs targeting different regions of *Slx2* were individually injected into mouse zygotes together with Cas9 mRNA. F1 mutant mice were obtained within 4–5 months. Eleven out of fifteen lines of F1 mutant mice did not express SLX2 anymore. However, the *Slx2* knockout mice were fertile. In-depth analysis of testes, epididymis and sperm showed that *Slx2* knockout did not result in any abnormality. Taken together, our work proved that CRISPR/Cas9 system can speed up the process of *in vivo* gene function interrogation, especially testis specific X-linked genes, and *Slx2* is dispensable for mouse spermatogenesis.

## Materials and Methods

### Animals

C57BL/6 and CD1 mice were used in this study. Mice were housed under temperature-controlled (22 ± 1°C) and light-controlled (a light cycle of 14 h light: 10 h dark) conditions in specific pathogen-free (SPF) animal facility in Sun Yat-sen University. The Institutional Animal Care and Use Committee of Sun Yat-sen University, P.R.China approved all the experimental protocols concerning the handling of mice. CRISPR/Cas9 knockout mice were bred with wild-type mice for more than three generations to avoid off-target effects. Mice were genotyped from tail-snips and PCR product sequence analysis by using the Mouse Genotyping Kit (KAPA biosystems, KK7302) following the manufacturer’s protocol. PCR was performed using primers:

*Slx2*-T7E1-F: 5’-TTGTTTACCTGAGATCCACTAA-3’,

*Slx2*-T7E1-R: 5’-TTGATGAAGTTAGATCACCATT-3’.

### Semi-quantitative RT-PCR and real-time RT-PCR

Total RNA from mouse various tissues and testes at different stages of development were extracted using TRIzol reagent (Invitrogen, 15596–018). First-strand cDNAs were synthesized using RevertAid First Strand cDNA Synthesis Kit (Thermo Scientific, #K1621) according to the manufacturer’s instructions.

The sequences of the forward and reverse primers used in the Semi-quantitative RT-PCR assays were as follows:

*Beta-actin-*F: 5’-TTCTTTGCAGCTCCTTCGTTGCCG-3’,

*Beta-actin-*R: 5’-TGGATGGCTACGTACATGGCTGGG-3’,

*Slx2*-F: 5’-TCCTCGAAGGCCGCTGAA-3’,

*Slx2*-R: 5’-AACCCTGGGATGCTGAAAGTC-3’.

Semi-quantitative PCR was performed under the following cycling conditions using TAKARA Taq (TAKARA, R001A). Reaction mixture was first heated at 95°C for 3 min. 25 cycles were then carried out with the following parameters: denaturing at 95°C for 20 s, annealing at 60°C for 30 s, extension at 72°C for 45 s. Reaction was finished with a final extension at 72°C for 5 min.

The sequences of the forward and reverse primers used in the real-time RT-PCR assays were as follows:

*Gapdh*-q-F: 5’-CATGAGAAGTATGACAACAGCCT-3’,

*Gapdh*-q-R: 5’-AGTCCTTCCACGATACCAAAGT-3’,

*Slx2*-q-F: 5’-CAGCATCCCAGGGTTTTGC-3’,

*Slx2*-q-R: 5’-TCCAAGCATAACCTCCACCTT-3’,

*Plzf*-q-F: 5’-CTGGGACTTTGTGCGATGTG-3’,

*Plzf* -q-R: 5’-CGGTGGAAGAGGATCTCAAACA-3’,

*Sycp3*-q-F: 5’-AGCCAGTAACCAGAAAATTGAGC-3’,

*Sycp3*-q-R: 5’-CCACTGCTGCAACACATTCATA-3’.

Real-time PCR was performed under the following cycling conditions using SYBR green master mix (ABI, 4367659). Reaction mixture was first heated at 95°C for 10 min. 40 cycles were then carried out with the following parameters: denaturing at 95°C for 15 s, annealing and extension at 60°C for 60 s. Data were analyzed by The StepOnePlus™ System (ABI).

### Generation of rabbit anti-SLX2 polyclonal antibody

The entire coding region of mouse *Slx2* was subcloned into pENTR/D-TOPO vector (Invitrogen). The full length CDS was recombined to pDEST17 (Invitrogen) using LR reaction (Invitrogen, 11791–020). The 6×His fusion protein was expressed in *Escherichia coli* strain BL21 and purified using a Ni Sepharose High Performance column (GE Healthcare, 17-5268-01) according to the manufacturer's instructions under denatured condition and on column refolding. Purified protein was used to immunize two healthy rabbits (Shanghai Institutes for Biological Sciences, CAS). Antibody was purified from serum of the rabbits by affinity purification using an antigen-coupled column generated with AminoLink Plus Coupling Resin (Pierce, 20501).

### Western blot analysis

Tissues were homogenized in cold RIPA buffer (50 mM Tris-HCl pH8.0, 150 mM NaCl, 1% NP40, 1 mM EDTA pH8.0, 0.5% sodium deoxycholate, 0.1% SDS) supplemented with 0.2 mM phenylmethylsulfonyl fluoride and a protein inhibitor cocktail (Sigma, P8340). The homogenates were centrifuged at 15,000 rpm for 15 min at 4°C, and the sediment was discarded. Total protein concentration was measured with BCA Protein Assay Kit (Pierce, 23225). Protein lysates (20 μg) were separated in SDS-PAGE and electrotransferred to a nitrocellulose membrane (Millipore, HATF00010). The membrane was blocked with 5% BSA in PBS for 1 h at room temperature and incubated with the following primary antibodies at room temperature for another 1 h. The primary antibodies were used at the indicated dilutions: rabbit anti-SLX2 antibody (1: 2,000), mouse anti-GAPDH antibody (1: 8,000; Proteintech group, 60004-1-Ig). Goat anti-mouse IgG (1: 10,000; LI-COR, 926–32220) and goat anti-rabbit IgG (1: 10,000; LI-COR, 926–32211) were used as secondary antibodies. The membrane was scanned using the Licor Odyssey system.

### Generation of gRNAs expressing vector

Three gRNAs were designed using http://crispr.mit.edu. The sequences of the gRNAs are as follow:

gRNA1: catacaattatgtttccccgTGG;

gRNA2: gggtcccttgtggtgctttcAGG;

gRNA3: ttcactcctctgcaaaacccTGG.

The Cas9 and gRNA expression vector pX330-U6-Chimeric_BB-CBh-hSpCas9 was a gift from Feng Zhang [27] (Addgene plasmid # 42230). The linearized vector pX330 was ligated with these gRNAs following Feng Zhang’s protocol.

The gRNA expression vector pDR274, a gift from Keith Joung [33] (Addgene plasmid # 42250), was linearized and ligated with the gRNAs as described above.

### Cell culture and transfection

V6.5 mouse embryonic stem (ES) cells were cultured with standard ES cell culture conditions. The gRNA ligated pX330 vectors were transfected into V6.5 ES cells using P3 primary cell 4D-nucleofecton kit (Lonza, V4XP-3024) according to the manufacturer’s instructions.

### T7E1 assay

Genomic DNA was extracted from both transfected and control V6.5 mouse ES cells 48 h after transfection using DNeasy blood & tissue kit (Qiagen, 69506). PCR was performed using primers as follows:

*Slx2*-T7E1-F: 5’-TTGTTTACCTGAGATCCACTAA-3’,

*Slx2*-T7E1-R: 5’-TTGATGAAGTTAGATCACCATT-3’.

The cycling condition was as follow using TAKARA Taq (TAKARA, R001A). Reaction mixture was first heated at 95°C for 5 min. 35 cycles were then carried out with the following parameters: denaturing at 95°C for 30 s, annealing at 60°C for 30 s, extension at 72°C for 40 s. Reaction was finished with a final extension at 72°C for 5 min.

PCR products were then denatured at 95°C for 5 min, annealed by cooling down at room temperature for 30 min, and treated with T7 endonuclease I (NEB, M0302L) at 37°C for 15 min. Digested DNA was separated on a 3% agarose gel.

### Production of Cas9 mRNA and gRNA

The Cas9 expression vector MLM3613 was a gift from Keith Joung [33] (Addgene plasmid # 42251). Since the Cas9 in MLM3613 is zebrafish expression optimized, mammalian expression optimized Cas9 CDS was subcloned from pX330 to MLM3613. Then the vector was linearized and *in vitro* transcribed as mRNA using mMESSAGE mMACHINE T7 Ultra Kit (Ambion, AMB1345-5).

The gRNAs ligated pDR274 were linearized and *in vitro* transcribed into RNA using MEGAshortscript T7 Transcription Kit (Ambion, AM1354).

### One-cell embryo injection

C57BL/6 and CD-1 mouse strains were used as embryo donors and foster mothers, respectively. Superovulated female C57BL/6 mice (5–6 weeks old) were mated with C57BL/6 stud males, and zygotes were collected from the ampulla. 200 ng/μL Cas9 mRNAs and 100 ng/μL gRNA were mixed and then 10 pL of the mixture was injected into the cytoplasm of each zygote. Majority of the surviving zygotes after injection were transferred into oviducts of foster mothers, while the remaining embryos were cultured *in vitro* for genomic DNA extraction after one-day culture. Conditions of PCR were described above using the T7E1 assay primers.

### Histological analysis

Testes were fixed in Bouin’s solution, embedded in paraffin, sectioned in 2 μm, sequential rehydrated and stained with hematoxylin and eosin.

### Immunofluorescent analysis of spread cells

Testicular cell suspensions were prepared as Hiroshi Kubota and Ralph L. Brinster described [34] with minor modification. Cells were spin to slides with ddH_2_O using CYTOPRO cytocentrifuge. The slides were fixed in 4% PFA in PBS for 15 min at room temperature and washed with PBS. Samples were permeabilized with 0.5% Triton X-100 in PBS for 10 min at room temperature and washed with PBS. Slides were incubated with blocking buffer (5% BSA in PBS) for 30 min at room temperature. Primary antibodies were diluted in blocking buffer and incubated at room temperature for 1 h. Primary antibody dilutions are as follow: rabbit anti-53BP1 (1: 5,000; Novus, NB100-304), mouse anti-SYCP3 (1: 200; Santa Cruz Biotechnology, sc-74569). After primary antibody incubation, slides were washed in PBS three times for 5 min each time. Secondary antibodies were also diluted in blocking buffer and incubated at room temperature for 1 h. Secondary antibodies were Alexa Fluor 488-conjugated donkey anti-rabbit IgG (1: 500, life technologies), Alexa Fluor 555-conjugated donkey anti-mouse IgG (1: 500, life technologies). After secondary antibody incubation, slides were washed in PBS three times for 5 min each time. Slides were mounted with DAPI in vectashield and stored at 4°C. Images were captured with ECLIPSE Ti-E (Nikon).

### Immunofluorescent analysis of testes sections

Testes were fixed in Bouin’s solution at 4°C within 48 h, embedded in paraffin, sectioned in 2 μm onto slides. Slides were rehydrated by immersing in serial concentration of ethanol. Slides were microwaved for 10 min in citric buffer (10 mM citric acid monohydrate, 10 mM sodium citrate). Slides were washed in PBS three times and incubated with blocking buffer (5% BSA in PBS) for 30 min at room temperature. The following procedures were as described above. Primary antibodies and dilutions are as follow: rabbit anti-SLX2 antibody (1: 500), goat anti-PLZF antibody (1: 500; R&D systems, AF2944), mouse anti-SYCP3 (1: 200; Santa Cruz Biotechnology, sc-74569), mouse anti-γH2AX (1: 200; Millipore, 05–636). Secondary antibodies and dilutions were as follow: Alexa Fluor 488-conjugated donkey anti-mouse IgG (1: 500, life technologies), Alexa Fluor 488-conjugated donkey anti-goat IgG (1: 500, life technologies), Alexa Fluor 555-conjugated donkey anti-rabbit IgG (1: 500, life technologies).

### Epididymal sperm analysis

The cauda epididymis was dissected from adult mice. Sperm was squeezed out from the cauda epididymis into 37°C 3% BSA (Sigma) in DMEM/F-12 (Gibico, 11039–021). The squeezed sperm was diluted with ddH_2_O for counting with a hemocytometer or diluted with 3% BSA in DMEM/F-12 for motility analysis using computer assisted sperm analysis system (Sperm Class Analyzer, MICROPTIC S.L.).

### Analysis of apoptotic cells

Annexin V/PI assay were performed on testicular cell suspensions which prepared as described above following the manufacturer's instructions (Beyotime Biotechnology, C1062). Flow cytometry analyses were performed using FACS Calibur (BD, 352235). TUNEL assay was performed according to the manufacturer’s instruction (Roche, 11684795910).

### Analysis of different ploidy of cells

Testicular cell suspensions were prepared as described above. Cells were centrifuged at 1,000×g for 2 min and washed with PBS for 3 times. 10^6^ to 10^7^ cells were resuspended with 300 μL PBS and 700 μL ethanol and vortex for 5 s. Samples were fixed at 4°C overnight. Cells were centrifuged at 1,000×g for 2 min and washed with PBS for 3 times and resuspended with 50 μg/mL RNase A in PBS then incubated at 37°C for 10 min. Prodium iodide was added to the cells to final concentration of 50 μg/mL. Flow cytometry analyses were performed using FACS Calibur (BD).

### Statistical analysis

All data are presented as the mean ± SD. The statistical significance of the difference between the mean values for the different genotypes was examined using unpaired two-tailed *t*-test. Quantitive PCR results were analyzed by applying one way ANOVA and Holm-Sidak test using SigmaPlot version 12.5. The data were considered significant when P < 0.05 (*), 0.01 (**) or 0.001 (***). Otherwise, it would be considered not significant (N.S.).

## Results

### Specific expression of SLX2 in mouse testis

**Fig 1:**
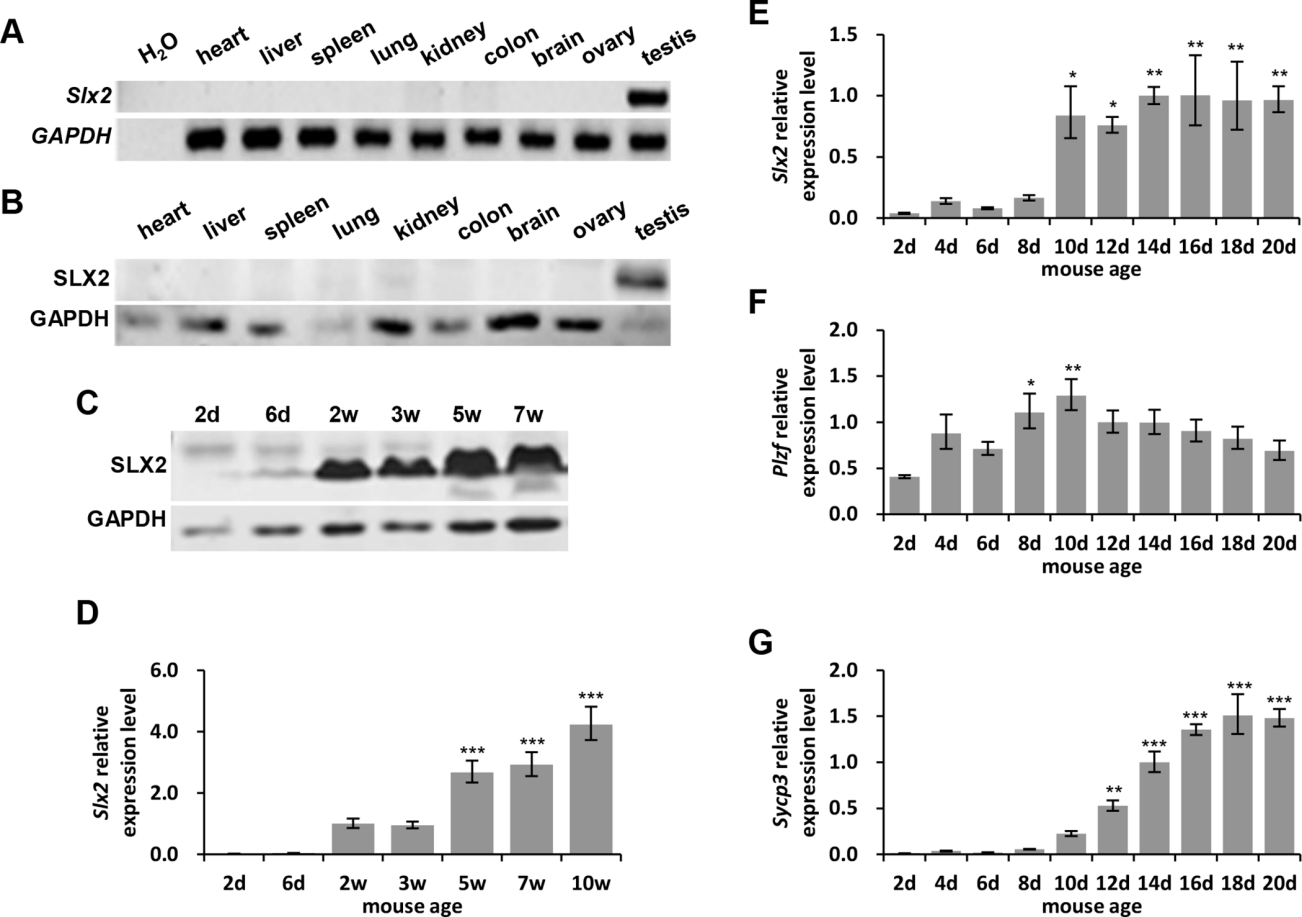
Specific expression of SLX2 in mouse testis. A) Semi-quantitative RT-PCR analysis of *Slx2* in different tissues from 3 weeks old mice. B) Western blot analysis of SLX2 in different tissues from 3 weeks old mice. C) Western blot analysis of SLX2 in testis from mouse of different ages. D) Quantitative real-time PCR analysis of *Slx2* in testis from mouse of different ages. E)–G) Quantitative real-time PCR analysis of *Slx2* (E), *Plzf* (F) and *Sycp3* (G) expression levels in mouse testes during the first wave of spermatogenesis. d, days; w, weeks. Quantitative real-time PCR gene expression level was normalized by *Gapdh* level. Data were analyzed by applying one way ANOVA and Holm-Sidak test. Expression levels were considered significantly different from the 2d group when P < 0.05 (*), 0.01 (**) or 0.001 (***).

To identify the expression pattern of *Slx2* in mice, we examined mRNA and protein expression in nine different tissues from 3 weeks old wild type C57BL/6 mice. We found that *Slx2* only expressed in testis (Fig 1A and 1B). *Slx2* mRNA and protein expression level were up regulated with age in testis (Fig 1C and 1D). Both mRNA and protein started to be detectable in 2 weeks old mice and expression level notably increased in 5 weeks old mice. Having considered that gene functions in mouse testis could be speculated from its expression pattern during the first wave of spermatogenesis in the newborn mouse [35], we detected *Slx2* mRNA expression level throughout the first wave of spermatogenesis during the first 20 days after birth to find out at what stage of spermatogenesis *Slx2* plays a role. Expression pattern of *Slx2* was different from *Plzf* (promyelocytic leukaemia zinc-finger), a marker of undifferentiated and differentiating spermatogonia in testis [36–38], but similar to *Sycp3* (synaptonemal complex protein 3), a marker of synaptonemal complex [39, 40] (Fig 1E–1G). *Slx2* mRNA level significantly increased after neonatal day 10 and then became stable, which led us to speculate that it may play a role in meiosis. However, *Slx2* expression boosted a little bit earlier than *Sycp3*, which indicated that *Slx2* might be expressed before the formation of synaptonemal complex. These data suggested that expression level of *Slx2* start to be up regulated dramatically in meiotic prophase I.

### Generation of *Slx2* knockout mice using CRISPR/Cas9

**Fig 2:**
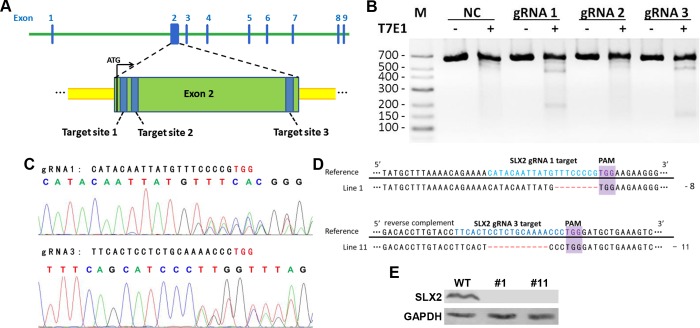
Generation of *Slx2* knockout mice. A) Schematic of the genomic target sites in the *Slx2* gene. B) Analysis of CRISPR/Cas9 system efficiency in mouse embryonic stem (ES) cells by using T7E1 assay. pX330 ligated with different gRNAs were transfected into V6.5 ES cells. Targeted region was PCR amplified and then digested by T7 Endonuclease I. M, marker. NC, negative control, mouse ES cells transfected with GFP plasmid. C) gRNA-1 and gRNA-3 worked in mouse embryos. Sequencing results of mouse embryos injected with Cas9 mRNA and *Slx2* gRNA-1 or gRNA-3 were shown. D) Genotyping results of line 1 and line 11 F1 mice. Cas9-mediated indels lead to frame shift of *Slx2*. Blue color showed the sequence of gRNAs; protospacer adjacent motifs (PAM) were labeled with purple color; red color showed the modified sequences information after targeting. -, nucleic acid base deletion. E) Western blot analysis of SLX2 expression level in testis from line 1 (#1) and line 11 (#11) mice. WT, wild-type.

By using CRISPR/Cas9 system, we generated founder mice within one month. Since *Slx2* locates on the X chromosome, we could produce knockout mice at the first generation (F1). First, we designed three gRNAs targeting different sites of the second exon of *Slx2* (Fig 2A). Both gRNA 1 and gRNA 3 could target *Slx2* effectively in V6.5 mouse ES cells indicated by T7E1 assay (Fig 2B). Then gRNA1 or gRNA3, together with Cas9 mRNA, were injected into mouse zygotes. The target region was amplified by PCR from five embryos for each gRNA and the PCR products were sequenced (Fig 2C). Sequencing results showed that both gRNA1 and gRNA3 could lead Cas9 to cleave target sites in mouse embryos. The rest of the CRISPR/Cas9 injected zygotes were transplanted into foster mothers after injection. Twenty founder mice were generated and fifteen of them were targeted by CRISPR/Cas9 and repaired with NHEJ (S2 Fig). Founder mice were then bred with wild type C57BL/6 mice to generate F1 mice. Eighteen lines with different genotypes were confirmed (S3A Fig). Then we detected SLX2 expression in testis by western blot. Result showed that eleven out of the fifteen out-frame mutant lines did not express SLX2 (S3B Fig). For further study, we chose two *Slx2* knockout lines for phenotype analysis (Fig 2D and 2E).

### Normal morphology of testis in *Slx2* knockout mice

**Fig 3:**
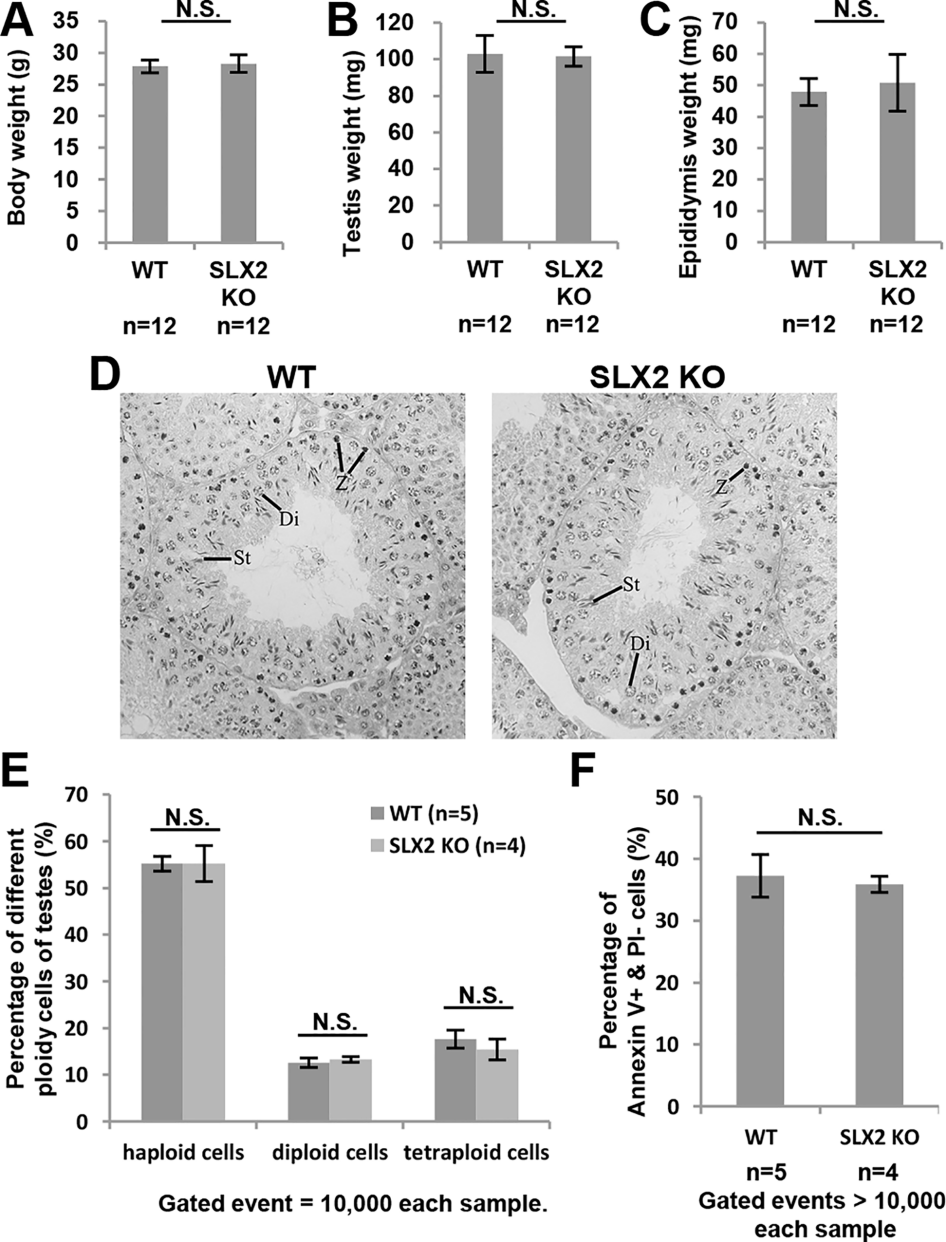
Normal morphology of testis in *Slx2* knockout mice. A)–C) Body weight, testis weight and epididymis weight analysis of 16 weeks old male mice. D) H&E staining of paraffin embedded 14 weeks old mouse testes section. Stage XI seminiferous tubule cross section was shown. No conceivable differences could be observed. Z, zygotene spermatocytes; Di, diplotene spermatocytes; St, elongating spermatids. E) Percentage of different ploidy cells in 7 weeks old mice testes was shown. F) Using Annexin V/PI staining flow cytometry to detect percentage of Annexin V positive and PI negative cells in 7 weeks old mice testes. N.S., not significant; WT, wild-type.

Since SLX2 was expressed in testis specifically, we analyzed the phenotype of *Slx2* knockout mouse specially focusing on spermatogenesis. Body weights of 16 weeks old *Slx2* knockout mice (28.28 ± 1.38 g) were almost the same as wild-type mice (27.88 ± 1.01 g) (Fig 3A). There was also no significant difference in testes weights between *Slx2* knockout mice (101.48 ± 5.26 mg) and wild-type mice (102.88 ± 10.07 mg) (Fig 3B). Additionally, weights of epididymides in *Slx2* knockout mice (50.88 ± 9.07 mg) and wild-type mice (47.90 ± 4.33 mg) were not distinguishable (Fig 3C). After normalization by comparing the testis weight or epididymis weight to body weight ratio, no significant difference between the two groups could be observed, neither. Testis weight to body weight ratio of *Slx2* knockout mice was 0.36 ± 0.02% and the ratio of wild-type mice was 0.37 ± 0.04%. Epididymis weight to body weight ratio of *Slx2* knockout mice was 0.18 ± 0.03% and the ratio of wild-type mice was 0.17 ± 0.02%. Hematoxylin and eosin (H&E) staining of testis section from 14 weeks old mice showed that there was no structural abnormality of *Slx2* knockout mouse testis comparing with wild-type mouse testis (Fig 3D). Proportion of haploid, diploid and tetraploid cells of 7 weeks old *Slx2* knockout mouse testis were similar to the wild-type mouse (Fig 3E), suggesting that the meiosis process might be not affected by *Slx2* knockout. During spermatogenesis, apoptosis plays a very important role in quality control of sperm production [41]. We further analyzed the percentage of apoptotic cells in testis using Annexin V/PI assay and TUNEL assay. Percentage of Annexin V positive and PI negative cells in wild-type testis and *Slx2* knockout testis were similar (Fig 3F). Having considered the high apoptotic ratio of Annexin V/PI assay might be caused by rapid elimination of dying cells by phagocytosis [42] and generation of dying cells during the process of preparation of cell suspension, we also performed TUNEL assay on testis sections. TUNEL results showed no significant difference between wild-type and *Slx2* knockout male mice (S4 Fig). Therefore, disruption of *Slx2* did not affect testis morphology.

### *Slx2* knockout did not affect cell distribution and XY body formation

**Fig 4:**
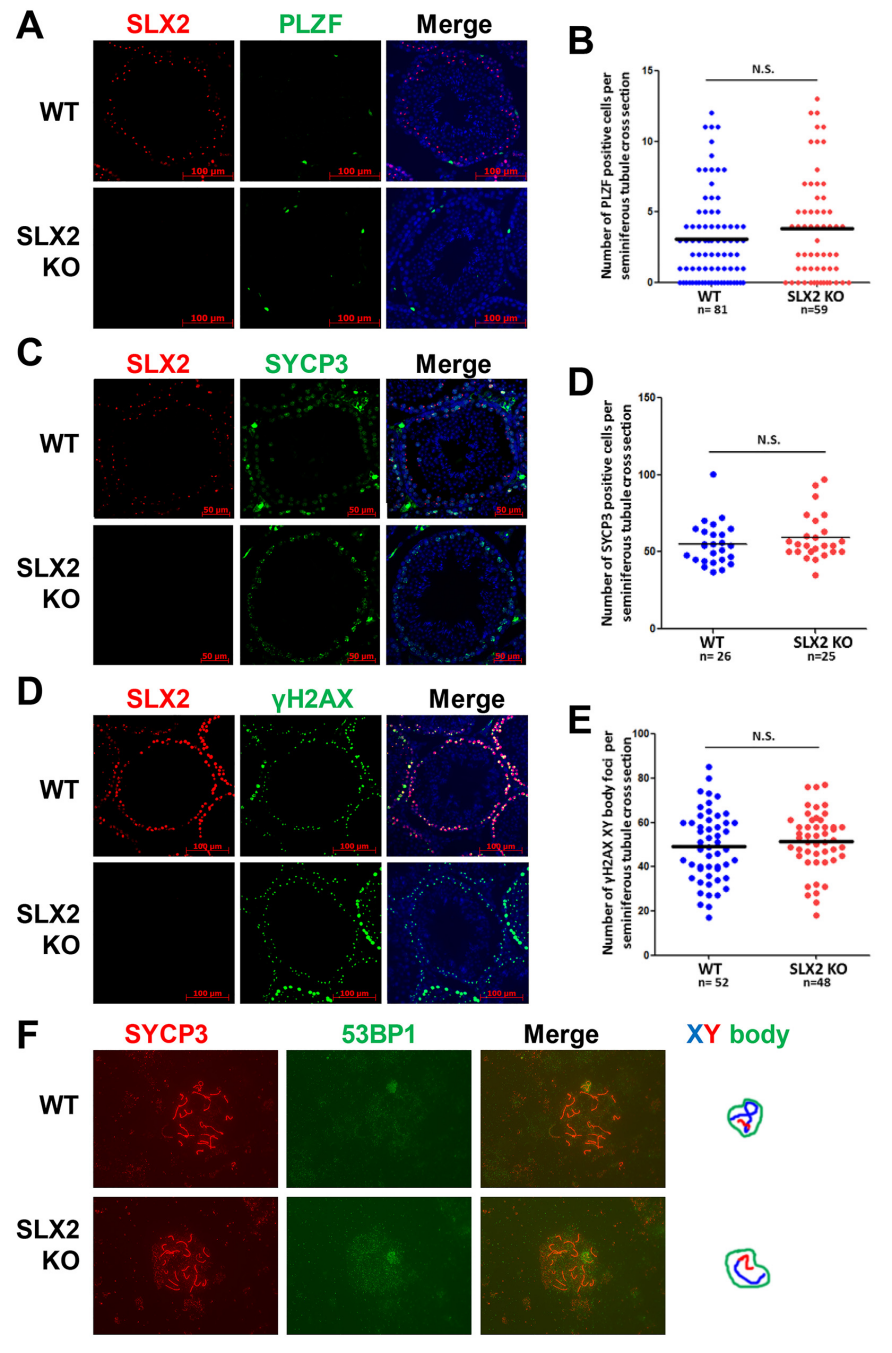
*Slx2* knockout did not affect cell distribution and XY body formation. A)–E) Immunofluorescence staining of 14 weeks old male mice paraffin embedded testes sections and quantification. PLZF is a spermatogonia stem cell marker. SYCP3 is a marker of synaptonemal complex. γH2AX forms specific foci at pachytene cells. SLX2 was labeled in red; PLZF, SYCP3 and γH2AX were labeled in green; cell nuclear labeled by DAPI was in blue. F) Immunofluorescence staining of 53BP1, which indicates the XY body in pachytene cells. No conceivable abnormality can be observed. SYCP3 was labeled in red; 53BP1 was labeled in green. X chromosome was drawn in blue; Y chromosome was drawn in red; the XY body was outlined with green. N.S., not significant; WT, wild-type.

To investigate SLX2 function in depth, we analyzed different cell types in testis from 14 weeks old mice. First, we focused on the analysis of spermatogonial stem cells (SSCs) and meiotic prophase cells. We quantified the number of these cells in seminiferous tubule by immunofluorescence staining of SSC marker PLZF (promyelocytic leukaemia zinc-finger) [36–38], meiosis synapsis marker SYCP3 (synaptonemal complex protein 3) [39, 40] and pachytene cell XY body marker γH2AX [43–45]. Numbers of PLZF, SYCP3 and γH2AX foci cells per seminiferous tubule cross section were quantified and showed no significant differences between wild-type male mice and *Slx2* knockout male mice (Fig 4A–4E), which proved that SLX2 itself is not required for homeostasis of SSCs and meiotic prophase cells. Previously studies found that SLX2 formed foci at the XY body [6, 7]. Many DNA damage response proteins locate at the XY body [5]. Here, we chose 53BP1 as an indicator of the XY body. Disruption of *Slx2* did not affect the formation of the XY body (Fig 4D, 4E and 4F).

### *Slx2* knockout mice produced healthy sperm and were fertile

**Fig 5:**
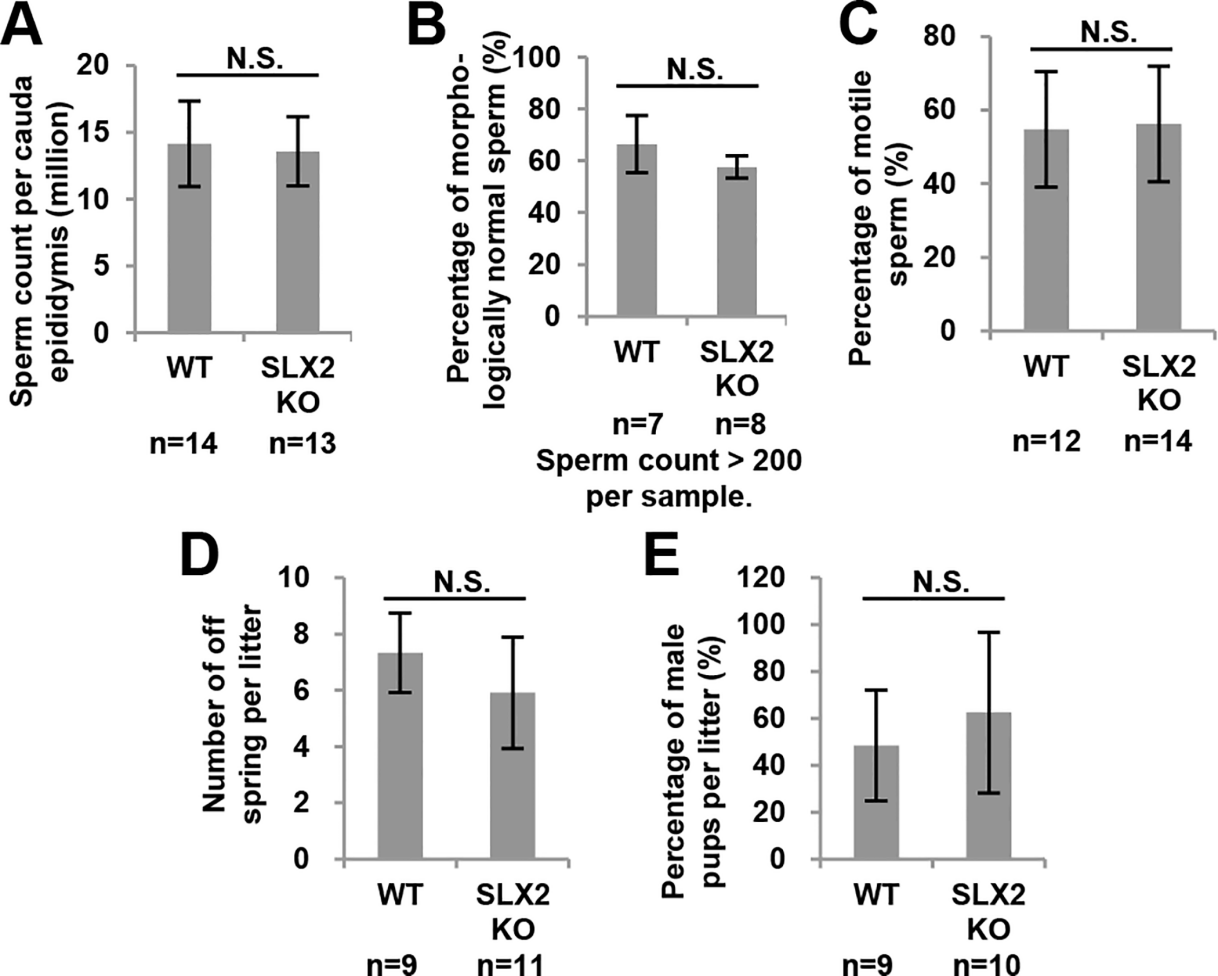
Sperm analysis and fertility test of male *Slx2* knockout mice. A) Sperm number per cauda epididymis of 16 to 21 weeks old mice. B) Percentage of morphologically normal sperm in 16 to 21 weeks old mice. C) Percentage of motile sperm in 16 to 21 weeks old mice. D) Number of off spring per litter. E) Percentage of male pups per litter. N.S., not significant; WT, wild-type.

Since the *Slx2* knockout did not affect spermatogenesis at the mitosis and meiosis stage, we try to figure out if disruption of *Slx2* affects later stage of spermatogenesis. We found that *Slx2* knockout did not affect quantity of sperm (Fig 5A). There were 14.59 ± 3.29 million sperms per cauda epididymis in wild-type mouse and 14.53 ± 1.53 million sperms per cauda epididymis in *Slx2* knockout mouse (Fig 5A). Depletion of SLX2 also did not affect the morphology of sperm and the motility of sperm (Fig 5B and 5C).

*Slx2* knockout spermatozoa have normal motility parameters (Table 1). Velocity of curve line, straight line velocity, average path velocity, linearity, straightness, wobbliness, amplitude of lateral head displacement, beat cross frequency of *Slx2* knockout spermatozoa were similar to wild-type spermatozoa. These data suggested that motility of *Slx2* knockout spermatozoa was not defected.

**Table 1:** CASA analysis of sperm motility parameters.

Parameter	Wild-type (n = 12)	SLX2 KO (n = 14)	*p* Value
Velocity of curve line (μm/s)	227.20 ± 51.22	213.63 ± 52.12	0.51
Straight line velocity (μm/s)	72.68 ± 18.59	68.08 ± 19.94	0.55
Average path velocity (μm/s)	110.76 ± 27.93	104.07 ± 28.33	0.55
Linearity (%)	32.00 ± 3.45	32.07 ± 5.25	0.97
Straightness (%)	65.77 ± 3.76	65.74 ± 7.18	0.99
Wobbliness (%)	48.56 ± 3.55	48.55 ± 3.61	0.99
Amplitude of lateral head displacement (μm)	9.67 ± 2.95	9.40 ± 3.28	0.83
Beat cross frequency (Hz)	6.04 ± 1.93	6.39 ± 2.06	0.66

With healthy sperm, *Slx2* knockout male mice could generate offspring normally (Fig 5D) which indicated that disruption of *Slx2* did not affect fertility of male mice. And sex ratio of *Slx2* knockout mice offspring were no different (Fig 5E). Our data showed that disruption of *Slx2* does not affect spermatogenesis and the function of mature sperm.

## Discussion

Though mouse spermatogonial stem cells (SSCs) can be cultured *in vitro* [34] and sperm can be produced *in vitro* [46–48], it is very difficult to study spermatogenesis *in vitro* due to the complexity of the platform and relatively low efficiency. Another way to investigate the functions of a gene in spermatogenesis is to generate gene modified mouse models. However, generating knockout mice using traditional gene targeting method is time-consuming and cost-intensive. Due to the emergence of CRISPR/Cas9 system, scientists can produce knockout mice much more quickly, easily and cost-effective at present.

*Slx2* is a testis specific X-linked gene so that we can generate *Slx2* knockout male mice by CRISPR/Cas9 in a very short time. Although previous data suggested that SLX2 might play an important role in spermatogenesis [6, 7], *Slx2* knockout male mice were fertile (Fig 5D), and we could not detect any defects during spermatogenesis (Figs 3 and 4). These data indicate that *Slx2* is not an essential gene for mouse spermatogenesis. It was reported that SLX2 was also expressed during mouse oocyte meiotic maturation [6, 49], but *Slx2* knockout female mice were fertile with normal number of offspring (unpublished data), which suggested that *Slx2* itself might be a non-essential gene for mouse oogenesis.

Numerous genes mutated or disrupted in mice have been reported to affect spermatogenesis. However, there are still some genes that are not as important as the others. There is a bunch of explanations to tell why a knockout mouse does not have any “phenotype”, if only the famous “Every knockout has a phenotype.” is true. Helen Pearson had discussed about this topic in a very comprehensive way [50]. Sometimes different phenotypes can be observed if the researchers change the mouse strain or expose the mouse to particular environmental conditions. Sometimes the gene function is covered up by the redundancy of the genes or by parallel pathways.

In our study, we found that CRISPR/Cas9 technique could accelerate our progress in functional gene functions in mouse models, especially those tissues specific sexual chromosome genes.

## Supporting Information

S1 Fig Verification of SLX2 antibody. Western blot analysis of SLX2-pBabe-CMV-SFB HTC75 stable cell line, which expressed SLX2—S tag—Flag tag–SBP tag fusion protein. 1, non-treated; 2, *Slx2* siRNA treated. IB, immunoblotting. Black arrow indicated SLX2 protein.
(TIF)

S2 Fig Sequencing results of twenty F0 mice. Double peaks showed that the two alleles of *Slx2* were different. Red arrows indicated base pair deletion in both alleles. Sequencing information of F0-16, F0-17, F0-18, F0-19 and F0-20 mice were no different from wild-type.
(TIF)

S3 Fig Genotype analysis of different *Slx2* F1 mice. A) Sequencing results of F1 mice. Blue color showed the sequence of gRNAs; protospacer adjacent motifs (PAM) were labeled with purple color; red color showed the modified sequences information after targeting. -, nucleic acid base deletion. B) Western blot analysis of the expression of SLX2 in different lines of F1 mice. WT, wild-type. The number indicated the line number of mice as showed in A). Liver used as negative control.
(TIF)

S4 Fig Detection of apoptotic cells in testis with TUNEL assay. A) Testis sections of 3 weeks old wild-type mice. B) Testis sections of 3 weeks old *Slx2* knockout mice. Green indicates TUNEL signal. Blue indicates DAPI signal.
(TIF)
