# Supplementary figures and images for: CRISPR/Cas9 Promotes Functional Study of Testis Specific X-Linked Gene In Vivo
Source: PLoS One. 2015 Nov 24;10(11):e0143148. doi: 10.1371/journal.pone.0143148 (PMC4658030; doi:10.1371/journal.pone.0143148)

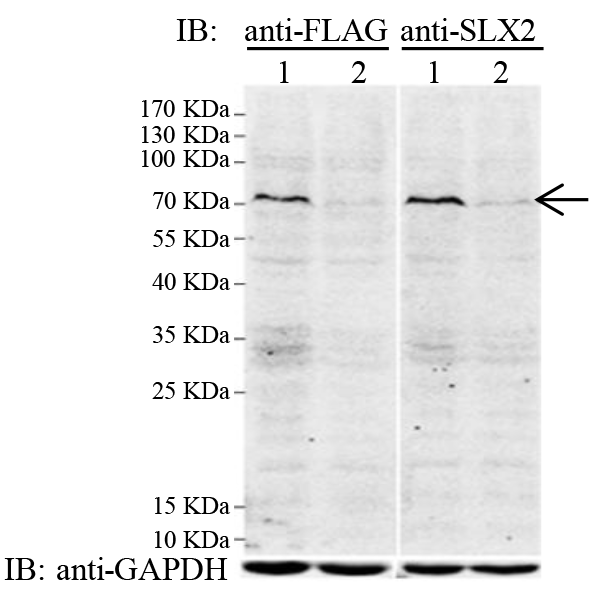

Supplement: S1 Fig — Western blot analysis of SLX2-pBabe-CMV-SFB HTC75 stable cell line, which expressed SLX2—S tag—Flag tag–SBP tag fusion protein. 1, non-treated; 2, Slx2 siRNA treated. IB, immunoblotting. Black arrow indicated SLX2 protein. (TIF) [file pone.0143148.s001.tif]

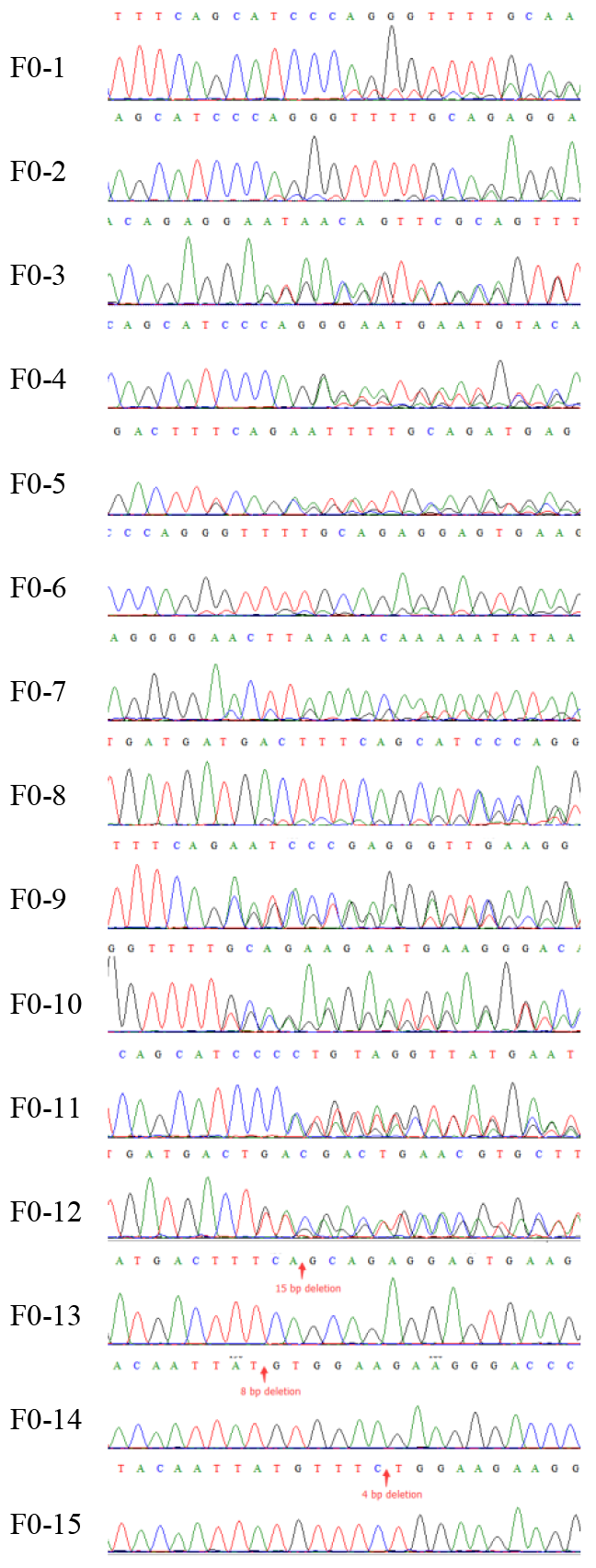

Supplement: S2 Fig — Double peaks showed that the two alleles of Slx2 were different. Red arrows indicated base pair deletion in both alleles. Sequencing information of F0-16, F0-17, F0-18, F0-19 and F0-20 mice were no different from wild-type. (TIF) [file pone.0143148.s002.tif]

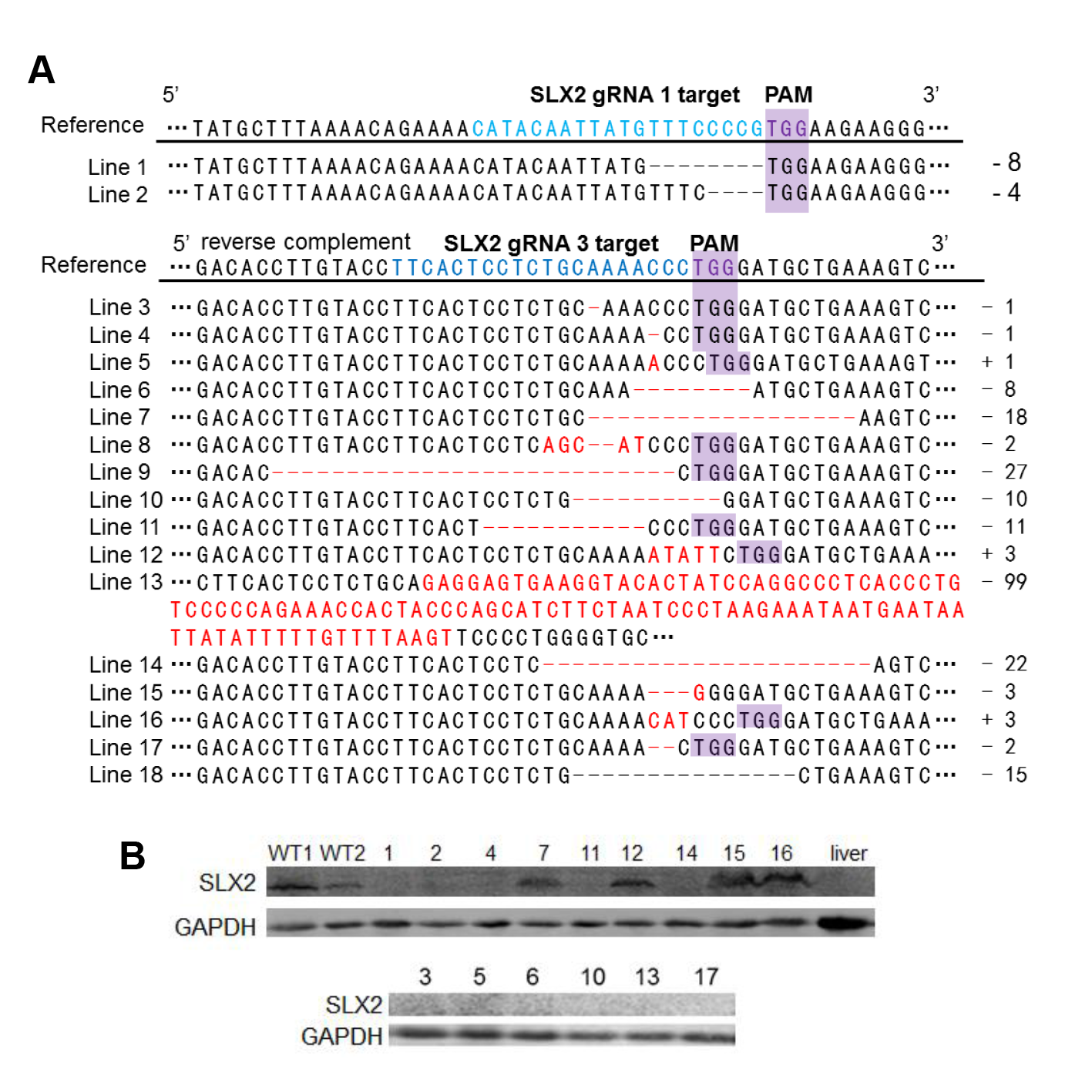

Supplement: S3 Fig — A) Sequencing results of F1 mice. Blue color showed the sequence of gRNAs; protospacer adjacent motifs (PAM) were labeled with purple color; red color showed the modified sequences information after targeting. -, nucleic acid base deletion. B) Western blot analysis of the expression of SLX2 in different lines of F1 mice. WT, wild-type. The number indicated the line number of mice as showed in A). Liver used as negative control. (TIF) [file pone.0143148.s003.tif]

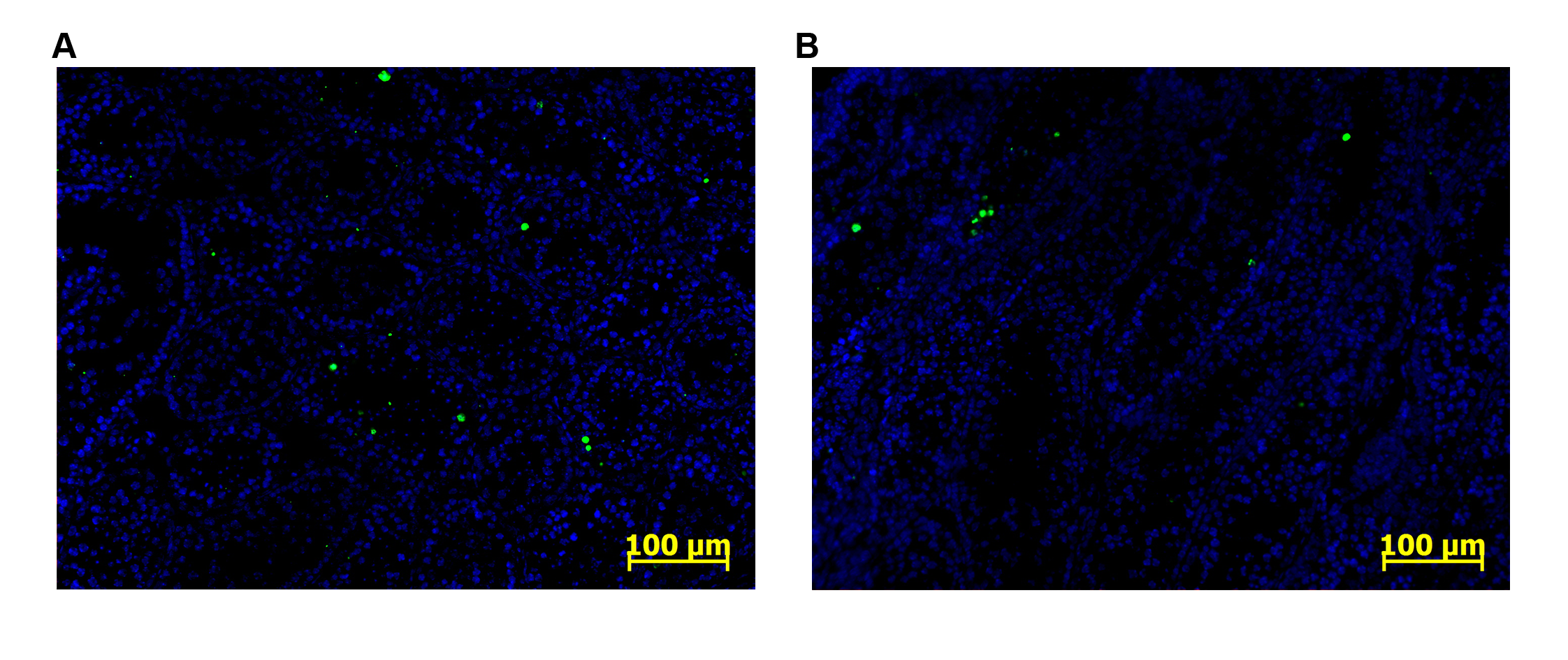

Supplement: S4 Fig — A) Testis sections of 3 weeks old wild-type mice. B) Testis sections of 3 weeks old Slx2 knockout mice. Green indicates TUNEL signal. Blue indicates DAPI signal. (TIF) [file pone.0143148.s004.tif]
